# Improved Healing after Non-Surgical Periodontal Therapy Is Associated with Higher Protein Intake in Patients Who Are Non-Smokers

**DOI:** 10.3390/nu13113722

**Published:** 2021-10-22

**Authors:** David W. Dodington, Hannah E. Young, Jennifer R. Beaudette, Peter C. Fritz, Wendy E. Ward

**Affiliations:** 1 Department of Kinesiology, Faculty of Applied Health Sciences, Brock University, St. Catharines, ON L2S 3A1, Canada; david.dodington@mail.utoronto.ca (D.W.D.); hy19nt@brocku.ca (H.E.Y.); jbeaudette3@gmail.com (J.R.B.); drpeterfritz@me.com (P.C.F.); 2 Periodontal Wellness and Implant Surgery Clinic, Fonthill, ON L0S 1E5, Canada

**Keywords:** periodontitis, periodontal diseases, scaling and root planning, diet, dietary protein

## Abstract

The aim of this study was to determine whether a relationship between periodontal healing and protein intake exists in patients undergoing non-surgical treatment for periodontitis. Dietary protein intake was assessed using the 2005 Block food frequency questionnaire in patients with chronic generalized periodontitis undergoing scaling and root planing (*n* = 63 for non-smokers, *n* = 22 for smokers). Protein intake was correlated to post-treatment probing depth using multiple linear regression. Non-smoking patients who consumed ≥1 g protein/kg body weight/day had fewer sites with probing depth ≥ 4 mm after scaling and root planing compared to patients with intakes <1 g protein/kg body weight/day (11 ± 2 versus 16 ± 2, *p* = 0.05). This relationship was strengthened after controlling for baseline probing depth, hygienist and time between treatment and follow-up (10 ± 2 versus 16 ± 1, *p* = 0.018) and further strengthened after controlling for potential confounders including age, sex, body mass index, flossing frequency, and bleeding on probing (8 ± 2 versus 18 ± 2, *p* < 0.001). No associations were seen in patients who smoked. Consuming ≥1 g protein/kg body weight/day was associated with reductions in periodontal disease burden following scaling and root planing in patients who were non-smokers. Further studies are needed to differentiate between animal and plant proteins.

## 1. Introduction

It is estimated that 70% of Canadians will experience periodontal disease in their lifetime [1]. Globally, the prevalence of periodontal disease is 11.2%, and severe periodontitis is the sixth most prevalent disease worldwide [2,3]. In the United States, the prevalence of periodontitis is 38% for adults over 30 years of age, and it increases to 64% in those over 65 years [4,5]. Periodontal disease has also been associated with numerous chronic illnesses including obesity, diabetes, metabolic syndrome, and rheumatoid arthritis [3,6,7]. While there are many known risk factors for periodontal disease, such as diet and smoking, there is a paucity of data on the roles of these factors in periodontal healing after scaling and root planing (SRP), the first-line non-surgical treatment modality for periodontitis [8]. Healing after SRP is dependent on the resolution of inflammation and reactive oxygen species and can result in reductions in probing depth (PD) and bleeding on probing (BOP) due to the repair and regeneration of damaged tissues [9,10]. SRP followed by routine periodontal maintenance appointments can prevent disease progression and ultimately, tooth loss. Tooth loss can have negative effects on speech, food intake, self-esteem and quality of life, and is independently associated with several chronic systemic inflammatory diseases [7]. Thus, it is important to understand the factors that may support periodontal healing post-SRP.

Smoking is a risk factor for numerous chronic diseases through increased inflammation and/or a compromised inflammatory response [11,12] and is a variable of interest due to its known negative effects on periodontal health [13]. Negative associations between smoking and periodontal bone loss, BOP, PD and clinical attachment loss have been found in patients following periodontal treatment [13,14,15]. While there is evidence to suggest diet is a risk factor for periodontal disease, this evidence is much weaker. Some associations are observed between periodontal health and nutrients with known anti-inflammatory, osteogenic and antioxidant activity [16,17,18,19,20,21]—these include calcium, vitamin C, flavonoids and omega 3 fatty acids. A previous study by our team reported associations between diet and healing post-SRP and found intakes of fruits and vegetables, β-carotene, vitamin C, α-tocopherol and long chain omega-3 fatty acids (EPA, DHA) to be positively associated with periodontal healing in patients who did not smoke [18]. There was no association between these same components in individuals who currently smoked [18]. It was hypothesized that the lack of an association was due to the negative effect of smoking on the periodontium, though it may also have been due to a small sample size.

With the paucity of data on protein intake and periodontal healing, protein was the primary nutrient of interest in the present study. Periodontal disease is most prevalent in adults over 65 years of age and has been associated with age-related diseases [22,23]. This, along with the fact that protein is the most important macronutrient for supporting immune function and wound healing [24], provided a rationale to determine if there is an association between higher protein intakes and periodontal healing. Of note, several organizations advocate that protein intakes of 1.0 to 1.5 g/kg body weight/day rather than the current recommended dietary allowance (RDA) for protein intake (0.8 g/kg body weight/day) are more appropriate for older adults (≥65 years) to reduce sarcopenia and its resultant loss in quality of life [25,26,27]. Sarcopenia is defined as a reduction in muscle mass and muscle function that occurs with aging [25,26]. The destruction of alveolar bone tissue is a prominent feature of periodontal disease; sufficient protein intake is associated with higher bone mineral density and slower rates of bone loss, again supporting a potential role of protein in periodontal healing [21,27,28]. Protein also has known anabolic effects on bone and may increase calcium absorption from the gut, leading to increased bone mineralization [28]. Moreover, two recent studies suggest a significant relationship between increased dairy consumption and decreased prevalence of periodontal disease [29,30]. Finally, a study in a rat model of periodontal disease found that consuming a higher level of milk basic protein (MBP) (1% versus 0.2%) supported greater recovery of alveolar bone that was lost as a result of previous ligature placement [31]. 

Given the widespread interest in higher protein intakes and health and indirect evidence suggesting a potential benefit in periodontal healing, the primary objective of this study was to determine whether the intake of dietary protein (≥1 g/kg body weight/day) was associated with a reduction in periodontal disease burden (i.e., fewer sites with PD ≥ 4 mm) following non-surgical treatment of periodontitis.

## 2. Materials and Methods

### 2.1. Study Population and Design

The data presented in this study are a retrospective analysis from a patient cohort initially investigated to study the relationship between fruits and vegetables, antioxidants, vitamins and omega-3 fatty acid intake and healing following SRP [18]. The study took place at a specialty periodontal clinic (Fonthill, ON, Canada) between January 2013 and July 2014. All patients undergoing SRP during this time were invited to participate in the study. Patients were excluded from the study analysis if they did not meet the requirements for chronic generalized periodontitis set by the American Academy of Periodontology (PD of ≥4 mm in at least 30% of sites) [32]. While a new periodontal classification system was developed in 2017, after this study was conducted, and the term ‘chronic periodontitis’ is no longer used, reporting the number and proportion of teeth with PD ≥ 4 mm remains a clinically meaningful periodontal measure in the new classification system [33]. During the enrolment visit, a comprehensive baseline dental examination including medical/dental history and periodontal charting was completed (see Section 2.2, Periodontal Examination, for more details). Full mouth SRP was then performed by one of four calibrated hygienists using hand and ultrasonic instruments as necessary. Individualized oral hygiene instructions were also provided to all patients. Participants were provided with a food frequency questionnaire (FFQ) to be completed at home and returned at the follow-up appointment. Follow-up occurred between 8 and 16 weeks after SRP and included a complete periodontal examination. The Bioscience Research Ethics Board at Brock University approved the study protocol and all participants provided written informed consent. This trial was registered at clinicaltrials.gov (accessed on 18 October 2021) as NCT02291835.

### 2.2. Periodontal Examination 

Baseline and follow-up periodontal charting included measurement of PD, BOP and plaque index (PI). PD was measured using a periodontal probe as the distance from the gingival margin to the bottom of the periodontal pocket at six sites per tooth (mesiobuccal, buccal, distobuccal, mesiolingual, lingual, and distolingual) on all teeth present, and the number of sites ≥4 mm were counted. Teeth to be extracted were not included in the count. BOP and PI were assessed by visual inspection after probing, and PI was measured using the O’Leary Plaque Index [34]. The baseline examinations were all performed by the same periodontist (P.C.F.) and occurred 1 to 19 weeks prior to SRP. Follow-up examinations were performed by one of four hygienists at 8 to 16 weeks post-SRP. Prior to the study, the hygienists were calibrated to apply 25 N of pressure when probing by repeated probing simulations against an electronic scale.

### 2.3. Diet Assessment

Nutrient intakes were assessed using the 2005 Block FFQ, which has been previously validated against multiple diet records [35] (https://www.nutritionquest.com/assessment/list-of-questionnaires-and-screeners/ (accessed on 21 October 2021)). The FFQ queried 110 food items for frequency (never, a few times a year, once per month, 2–3 times per month, once per week, 2 times per week, 3–4 times per week, 5–6 times per week, every day) and portion size. Portion size pictures were provided to enhance the accuracy of quantification. Nutrient intake estimates were calculated using a database of nutrient values derived from the Canadian Nutrient File. Assessment of reporting accuracy in this cohort has been previously published [18]. Nutrient intakes were energy adjusted and standardized to a 2000 Kcal diet using the residual method [36]. Protein intake was expressed in grams per kilogram of body weight and participants were classified by protein intake level (<1 g/kg body weight/day or ≥1 g/kg body weight/day). The 1 g/kg bodyweight/day cut-off was chosen due to evidence suggesting protein intakes ≥ 1 g/kg body weight/day may be more appropriate for older adults (>65 years) [25,26,27]. 

### 2.4. Assessment of Covariates 

Self-reported age, sex, health conditions, medication use and smoking status (never, former or current) were recorded from the patient’s medical history form. Dental hygiene information was gathered by a hygienist and included brushing and flossing frequency, electronic toothbrush use, frequency of professional dental cleaning and any previous periodontal therapy. Participants also met with a study nurse during their initial visit and had their height and body weight measured (Health-O-Meter Professional) to calculate their BMI. Blood was also drawn at this time for the measurement of serum 25-hydroxyvitamin D, which was analyzed by a third-party provider (Life Labs, Thorold, ON, Canada) using the Liaison chemiluminescence system (DiaSorin Inc., Mississauga, Canada). The laboratory participates in the Vitamin D External Quality Assessment Scheme (DEQAS).

### 2.5. Statistical Analysis 

Patients were stratified by smoking status for two main reasons: i. patients who smoke are known to have compromised healing after SRP [15,37] and ii. no associations between periodontal health and any of the previously analyzed nutrient intakes was previously observed in patients who smoke [18]. Descriptive statistics and nutrient intakes were compared between the protein intake groups using an independent sample t test for continuous variables and chi-square test for categorical variables. When the expected cell count was <5 for categorical variables, Fisher’s exact test was used in place of the chi-square test. Due to the small sample size and the violation of the assumption of normality in the group of patients who smoked, a Mann–Whitney U Test was used instead of the independent sample t test. The relationship between protein intake and periodontal healing was assessed by multiple linear regression using PD as a continuous dependent variable. Protein intake was entered into the model as a dichotomous variable (<1 versus ≥1 g protein/kg/day), first in an unadjusted model, then in subsequent models with adjustment for covariates. All models were assessed for normality of residuals and multicollinearity. Data were analyzed with SPSS v.20 (IBM, Inc., Armonk, NY, USA), and statistical significance was defined as *p* < 0.05.

## 3. Results

### Main Findings

**Figure 1:**
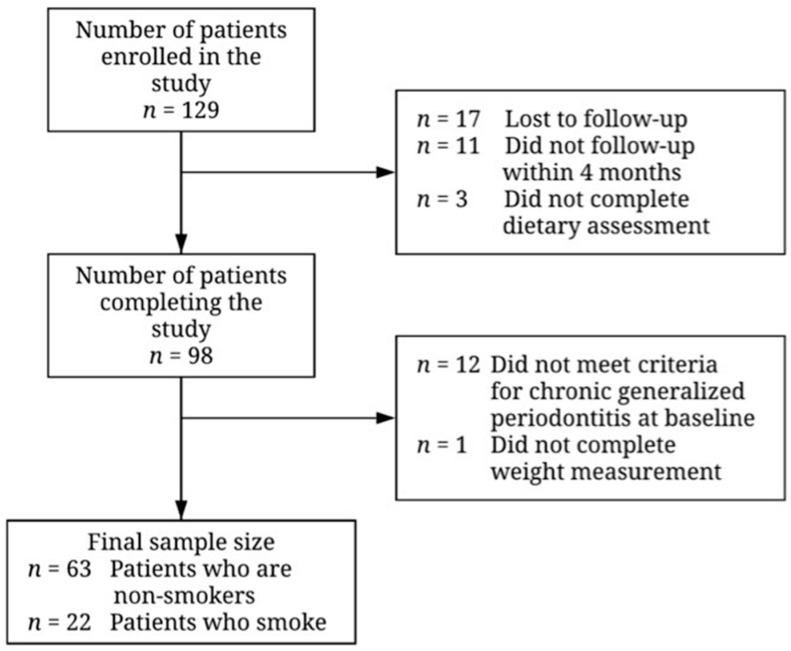
Flowchart illustrating recruitment and final sample size for patients undergoing non-surgical periodontal therapy. Reasons for exclusion of participants are given to the right of the arrows denoting the transitions between stages.

In total, 129 patients were recruited for the study (Figure 1). Of those, 17 patients were lost to follow-up, 11 did not return within the 16-week follow-up period, and 3 did not complete the food frequency questionnaire and were thus excluded from the analysis. Subsequently, 12 did not meet the criteria for chronic generalized periodontitis and were excluded. One additional patient was excluded as their body weight was not measured, so protein intake per kg of bodyweight could not be calculated. There were no differences in age, sex, BMI, smoking status, baseline clinical outcomes, or serum 25-hydroxyvitamin D concentration between those included and those excluded from the analysis (Table S1). 

Participant characteristics by level of protein intake and smoking status are shown in Table 1. Regardless of smoking status, participant age and comorbidities did not differ between the two levels of protein intake. For patients who were non-smokers, former smoking status did not differ based on protein intake. For both non-smokers and smokers, the ≥1 g/kg body weight/day protein group had significantly more female participants and they had a lower average BMI. In terms of dental hygiene, there were no differences in brushing frequency, electric toothbrush use, professional cleaning frequency and previous periodontal therapy based on protein intake levels in both the smoking and non-smoking groups. Patients who were non-smokers and consumed ≥1 g/kg/day of protein reported significantly higher flossing frequencies compared to those who consumed <1 g/kg body weight/day of protein; however, this difference was not observed in patients who smoked. 

Importantly, there were no differences in periodontal outcomes between protein intake groups at baseline, including the number of teeth, PD, BOP and PI, as well as the time between treatment and follow-up regardless of smoking status. By design, protein intakes differed between groups. Other nutritional intakes, including total energy, total carbohydrate, total fat, omega-3 fatty acid, vitamin C intakes and serum 25-hydroxyvitamin D were not significantly different between the two levels of dietary protein intake in those who did not smoke. In patients who smoked, only omega-3 fatty acid intake was significantly higher in those with protein intakes ≥1 g/kg body weight/day. 

**Figure 2:**
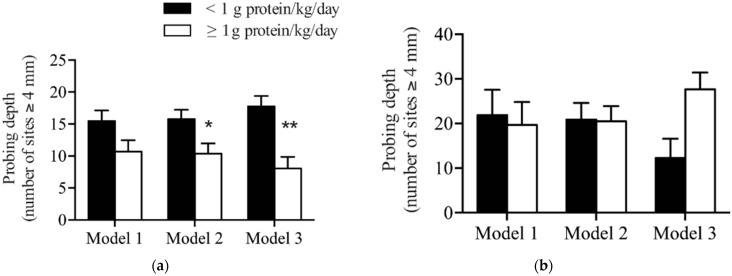
Comparison of dietary protein levels with probing depth after non-surgical periodontal therapy in patients who (**a**) did not smoke and (**b**) did smoke. Model 1 is unadjusted. Model 2 adjusted for baseline probing depth, hygienist and time between treatment and follow-up. Model 3 adjusted for variables in Model 2 plus age, sex, BMI, flossing frequency and bleeding on probing. Data presented are estimated marginal means ± SEM. * Indicates a significant difference between groups (*p* < 0.05), ** indicates a significant difference between the two levels of protein intake (*p* < 0.01).

In the unadjusted regression analysis, non-smoking patients who consumed ≥1 g/kg body weight/day of protein had fewer sites with PD ≥ 4 mm at the time of follow-up compared to those who consumed <1 g/kg/day of protein. This difference was borderline significant (11 ± 2 versus 16 ± 2, *p* = 0.05) (Model 1, Table 2 and Figure 2a). To improve the fit of the model, additional study design factors were considered in the regression model. This included the patient’s baseline PD, the hygienist performing their treatment and the time between treatment and follow-up. 

In this second model, patients who consumed ≥1 g/kg/day of protein had significantly fewer numbers of sites with PD ≥ 4 mm compared to patients who consumed <1 g/kg body weight/day (10 ± 2 versus 16 ± 1, *p* = 0.018) (Model 2, Table 2 and Figure 2a). Lastly, to control for potential confounders and to better understand the association between protein intake and PD, the confounders identified in Table 1 were added to the model: sex, BMI, flossing frequency, as well as age and BOP at follow-up as an objective measure of oral hygiene. 

In the fully adjusted model, there continued to be a significant association between the consumption of ≥1 g/kg body weight/day of protein and a reduction in the number of sites with PD ≥ 4 mm compared to patients who consumed <1 g/kg body weight/day (8 ± 2 versus 18 ± 2, *p* < 0.001) (Model 3, Table 2 and Figure 2a). In patients who smoked, no significant differences in the number of sites with probing depths ≥ 4 mm were observed based on protein intake in any of the models (Table 3 and Figure 2b).

## 4. Discussion

A key finding from this study is the association between higher consumption of dietary protein (≥1 g/kg body weight/day) and a greater reduction in the number of sites with a PD ≥ 4 mm in patients who were non-smokers after SRP. Moreover, this relationship was strengthened by controlling for baseline PD, hygienist and time between treatment and follow-up. It was even further strengthened after accounting for additional confounding factors such as age, sex, BMI, flossing frequency and BOP. While there is little literature available on the relationship between dietary protein and periodontal disease in non-smokers, that which is available supports the current findings. For example, feeding milk basic protein, containing whey protein and other potential bioactives, improved healing in a rat model of periodontal disease [31]. Periodontal disease was induced in rats by placing a ligature on the second maxillary molar for 20 days. The ligature was then removed, and varying levels of milk basic protein were added into the control diet at a level of 0.2% or 1.0% MBP while keeping the total level of protein similar among diets. The rats fed the diet containing a higher level of MPB (1.0%) had greater alveolar bone regeneration following 90 days compared to rats fed the control diet or the diet containing 0.2% MBP. 

Additional evidence supporting a relationship between dietary protein and periodontal disease comes from data from the fifth and sixth Korean National Health and Nutrition Examination Survey (*n* = 9798), which found that consuming ≥ 7 servings of dairy products per week was associated with a 24% lower prevalence of periodontal disease after controlling for confounding factors including calcium intake, BMI and smoking status [29]. Given that dairy foods tend to be high in protein, the authors hypothesized that whey proteins may prevent alveolar bone loss and attenuate periodontal disease. Similar findings were reported in a cohort study using Danish Health Examination Survey data (*n* = 3287) which found adults consuming ≥ 9.6 g of whey/day and/or ≥32 g casein/day had a lower likelihood of severe periodontal disease as defined by the American Academy of Periodontology [30,38]. 

A similar relationship between protein intake and the number of sites with PD ≥ 4 mm was not observed in patients who smoked. This may be a result of smoking having negative effects on oral health and healing—this was previously shown for other nutrients [18]. Smoking is one of the largest risk factors for periodontal disease and is thought to impact periodontal health through various mechanisms, including decreased vascular flow, decreased lymphocyte proliferation, and impairing neutrophil function, thus compromising wound healing [11,13,15,39]. However, it is important to note that there were relatively few patients who smoked within the study and as such, this specific analysis was underpowered but helps inform the design of future studies. Based on the results of this study, a retrospective power calculation was performed. Detecting a difference in the PD depth of 2.2 sites with a standard deviation of 18.0, (α = 0.05, β = 0.80) would require a sample size of *n* = 1050 per group, assuming a relationship exists in smokers. Given the small difference in PD seen in smokers, it is likely that either no relationship exists or that the effect size is too small to be clinically meaningful. 

It is possible that protein has both direct and indirect effects on periodontal health. One possible explanation of the observed relationship between probing depth and protein intake could be due to its direct effects on wound repair. For example, studies have found that protein malnutrition leads to delayed wound healing [40]. Moreover, in a randomized clinical trial of elderly home-nursed patients, consuming a nutrient- and protein-dense supplement resulted in improved wound healing [41]. The level and quality of protein intake are known to affect collagen deposition and other factors that result in delayed wound healing [39,40,41,42]. However, no studies have specifically studied protein intake and oral healing. Additionally, given that periodontal disease can result in a loss of bone and teeth, the osteogenic effects of protein are hypothesized to contribute to long-term periodontal health; however, these effects might not be expected to contribute directly to changes in probing depth during the 8 to 16 week follow-up. Studies of protein intake and bone health indicate that there is little benefit of increasing protein intake beyond levels of 0.8–1.3 g/kg body weight/day in healthy adults—and no detrimental effects of such higher protein intakes to bone mineral density or fracture risk provided calcium intake is at the recommended level [28]. However, levels of protein intake below 0.8 g/kg body weight/day could potentially have negative implications for bone health and periodontal disease. 

### Strengths and Limitations

One of the strengths of this study was that there were no significant differences in baseline periodontal outcomes between protein intake groups. Given that baseline PD was associated with periodontal outcomes 8 to 16 weeks post-SRP in our original study [18], this reduces the likelihood that baseline periodontal health served as a confounding factor in this study. Additionally, there were no differences in participant age, former smoking status, comorbidities, brushing frequency, electric toothbrush use, professional cleaning frequency and previous periodontal therapy between protein intake groups regardless of smoking status. The study also included approximately equal proportions of male and female participants. 

Some of the limitations are the use of FFQ to measure dietary intake, multiple hygienists performing periodontal examinations, variation in the length of time before SRP and follow-up time after SRP, and potential confounding variables such as dental hygiene practices. Due to the tendency of FFQs to overestimate food intakes [43], caution should be used when interpreting intake levels derived from this study and comparisons to the RDA. Additionally, the FFQ used was unable to differentiate between dairy, animal, and plant proteins. Thus, we are unable to draw specific conclusions based on protein type. The effects of multiple examiners were minimized by calibrating hygienists and performing statistical control for this variable. The variation in the length of time prior to and after SRP reflect the real-life situation of clinical practice, in which patients need to find time in busy schedules for these appointments. Baseline examinations occurred 1 to 19 weeks prior to SRP, and follow-up time ranged from 8 to 16 weeks, which was the standard of care at the time of the study. In patients who were non-smokers, flossing frequency was significantly higher in the group who consumed ≥ 1 g protein/kg body weight/day. While this may serve as a confounding factor, it is unlikely to have a large effect, given that all other oral hygiene variables showed no significant difference between groups, which suggests overall that patients had relatively similar oral hygiene behaviors. Additionally, including flossing frequency as a covariate in statistical models had minimal effect on the strength of the associations observed. The small sample size for the group of patients who were smokers is a limitation, though it was also encouraging that there were far fewer participants who smoked given the known detriment that smoking has on overall health.

## 5. Conclusions

This study shows a positive association between protein intakes ≥1 g/kg body weight/day and better periodontal healing following SRP in patients who do not smoke, but not in patients who smoke. Albeit there were few patients who were current smokers, so this association requires verification. Future dietary intervention studies during the post-SRP healing phase are warranted in order to determine the potential benefits of protein intake as an adjunct to SRP. Additionally, long-term studies should be carried out to measure the relationship between dietary protein intake, tooth loss and alveolar bone loss, as these measures may be better indicators of the long-term benefit of higher protein intakes for periodontal health.

## Figures and Tables

**Table 1:** Descriptive statistics and clinical parameters of patients undergoing non-surgical periodontal therapy grouped by protein intake and current smoking status ^1^.

	Patients Who Did Not Smoke			Patients Who Did Smoke		
	Protein Intake g/kg Body Weight/Day		*p* Value	Protein Intake g/kg Body Weight/Day		*p* Value
	<1	≥1		<1	≥1	
	*n* = 34	*n* = 29		*n* = 10	*n* = 12	
**Patient Characteristics**						
**Male**	25 (73%)	8 (28%)	**<0.001**	7 (70%)	2 (17%)	**0.027**
Female	9 (27%)	21 (72%)	**<0.001**	3 (30%)	10 (83%)	**0.027**
Age (years)	57 ± 10	61 ± 13	0.10	54 ± 8	52 ± 7	0.72
BMI (kg/m^2^)	31.8 ± 5.0	26.6 ± 3.8	**<0.001**	31.8 ± 6.1	24.6 ± 4.2	**0.004**
Former smokers	15 (44%)	18 (62%)	0.16	-	-	-
**Comorbidities**						
Diabetes	4 (12%)	2 (7%)	0.68	1 (10%)	1 (8.3%)	1.00
HTN or CAD	14 (41%)	10 (35%)	0.59	3 (30%)	2 (17%)	0.62
Osteoporosis	1 (3%)	5 (17%)	0.09	0	0	-
# of medications	3 ± 3	3 ± 4	0.75	2 ± 1	1 ± 2	0.25
**Dental hygiene**						
Brushing (times/day)	2.2. ± 0.6	2.4 ± 1.1	0.37	1.0 ± 0.7	2.3 ± 0.75	0.35
Flossing (times/week)	2.7 ± 4.4	5.3 ± 3.6	**0.016**	2.4 ± 4.7	5.0 ± 6.1	0.069
Electric toothbrush use	11 (32%)	10 (35%)	0.83	3 (27%)	3 (25%)	1.00
Cleanings (months)	5 ± 3	5 ± 3	0.54	5 ± 3	7 ± 6	0.67
Previous therapy	10 (29%)	8 (28%)	0.89	3 (30%)	3 (25%)	1.00
**Baseline clinical outcomes**						
Number of teeth	25 ± 4	25 ± 4	0.81	25 ± 4	26 ± 2	0.25
PD (# sites ≥ 4 mm)	93 ± 37	93 ± 33	0.96	102 ± 39	107 ± 34	0.77
BOP (# sites)	76 ± 46	77 ± 50	0.92	79 ± 61	73 ± 43	0.77
Plaque index (%)	78 ± 27	67 ± 30	0.19	83 ± 24	57 ± 37	0.080
Follow-up time (days)	74 ± 17	78 ± 15	0.37	79 ± 17	80 ± 16	0.92
**Follow-up clinical outcomes**						
Number of teeth	24 ± 4	24 ± 5	0.95	24 ± 4	26 ± 3	0.35
PD (# sites ≥ 4 mm)	16 ± 11	11 ± 8	0.05	22 ± 21	20 ± 15	1.00
BOP (# sites)	8 ± 9	3 ± 6	**0.009**	13 ± 18	4 ± 7	0.069
Plaque index (%)	36 ± 19	28 ± 25	0.14	47 ± 26	30 ± 25	0.50
**Nutritional intake and status**						
Calories (Kcal/day)	1625 ± 586	1583 ± 649	0.79	1828 ± 530	1525 ± 674	0.28
Protein (g/day)	79 ± 10	88 ± 9	**<0.001**	69 ± 13	84 ± 13	**0.025**
Carbohydrate (g/day)	244 ± 34	230 ± 37	0.14	231 ± 51	227 ± 33	0.72
Fats (g/day)	76 ± 12	78 ±13	0.43	77 ± 12	80 ±18	1.00
EPA + DHA (mg/day)	320 ± 463	380 ± 365	0.58	99 ± 61	253 ± 163	**0.021**
Vitamin C (mg/day)	130 ± 51	129 ± 37	0.90	105 ± 61	137 ± 73	0.25
25-OH-D (nmol/L)	59 ± 21	70 ± 25	0.074	44 ± 12	57 ± 21	0.159

^1^ All values are means ± SDs for continuous variables and counts (%) for categorical variables. Non-standard abbreviations: HTN, hypertension; CAD, coronary artery disease; PD, probing depth; BOP, bleeding on probing; EPA, eicosapentaenoic acid; DHA, docosahexaenoic acid; 25-OH-D, 25-hydroxyvitamin D.

**Table 2:** Multiple linear regression of probing depth sites > 4 mm in patients after non-surgical periodontal therapy who did not smoke ^1^.

	Model 1		Model 2		Model 3	
	*B* (95% CI)	*p*	*B* (95% CI)	*p*	*B* (95% CI)	*p*
Protein intake						
<1 g/kg body weight/day	Reference		Reference		Reference	
≥1 g/kg body weight/day	−4.8 (−9.6, 0.0)	0.050	−5.4 (−9.9, −1.0)	**0.018**	−9.7 (−15.5, −3.9)	**0.001**
Hygienist						
Hygienist 1			Reference		Reference	
Hygienist 2			−7.3 (−12.8, −1.8)	**0.011**	−7.2 (−12.4, −1.9)	**0.009**
Hygienist 3			−4.8 (−10.7, 1.0)	0.11	−6.1 (−11.9, −0.4)	**0.037**
Hygienist 4			−4.2 (−0.1, 0.2)	0.23	−6.3 (−13.7, 1.0)	0.09
Baseline PD (# ≥ 4 mm)			0.1 (0.01, 0.2)	**<0.001**	0.2 (0.1, 0.2)	**<0.001**
Follow-up time (days)			0.0 (−0.1, 0.2)	0.54	0.0 (−0.1, 0.2)	0.54
Sex						
Male					Reference	
Female					4.1 (−0.8, 9.0)	0.10
Age (years)					0.2 (0.0, 0.4)	**0.041**
BMI (kg/m^2^)					−0.5 (−1.0, 0.0)	**0.038**
Flossing (times/week)					0.0 (−0.5, 0.6)	0.89
BOP (# sites)					0.3 (0.0, 0.6)	0.09

^1^ Regression of probing depth (number of sites ≥ 4 mm) using low (<1 g/kg/day) and high (≥1 g/kg/day) protein intake as a categorical variable. Unstandardized regression coefficients (B) and 95% confidence intervals (CI) are shown. Non-standard abbreviations: BOP, bleeding on probing; PD, probing depth.

**Table 3:** Multiple linear regression of probing depth sites ≥ 4 mm after non-surgical periodontal therapy in patients who smoked ^1^.

	Model 1		Model 2		Model 3	
	*B* (95% CI)	*p*	*B* (95% CI)	*p*	*B* (95% CI)	*p*
Protein intake						
<1 g/kg body weight/day	Reference		Reference		Reference	
≥1 g/kg body weight/day	−2.2 (−18.3, 13.8)	0.77	−0.4 (11.9, 11.1)	0.95	16.3 (−1.4, 34.0)	0.066
Hygienist						
Hygienist 1			Reference		Reference	
Hygienist 2			−18.2 (−32.7, −3.8)	**0.017**	−25.0 (−44.7, −5.2)	**0.016**
Hygienist 3			−7.0 (−28.4, 14.4)	0.49	−9.8 (−35.3, 15.7)	0.45
Hygienist 4			−20.7 (−46.1, 4.8)	0.10	−26.2 (−55.2, 2.8)	**0.033**
Baseline PD (# ≥ 4 mm)			0.2 (0.0, 0.4)	**0.025**	0.1 (−0.2, 0.3)	0.38
Follow-up time (days)			0.3 (−0.1, 0.6)	0.17	0.0 (−0.5, 0.5)	0.98
Sex						
Male					Reference	
Female					−9.6 (−27.1, 7.9)	0.30
Age (years)					0.2 (−0.6, 0.9)	0.60
BMI (kg/m^2^)					0.3 (−1.0, 1.6)	0.66
Flossing (times/week)					−0.3 (−1.3, 0.7)	0.47
BOP (# sites)					0.8 (0.1, 1.4)	**0.017**

^1^ Regression of probing depth (number of sites ≥ 4 mm) using low (<1 g/kg/day) and high (≥1 g/kg/day) protein intake as a categorical variable. Unstandardized regression coefficients (*B*) and 95% confidence intervals (CI) are shown. Non-standard abbreviations: BOP, bleeding on probing; PD, probing depth.

## Data Availability

The data presented in this study are available upon request from the corresponding authors. The data are not publicly available due to ethics restrictions.

## Supplementary Materials

The following are available online at https://www.mdpi.com/article/10.3390/nu13113722/s1, Table S1: descriptive statistics and clinical parameters of excluded participants.
