# Supplementary material for: Improved Healing after Non-Surgical Periodontal Therapy Is Associated with Higher Protein Intake in Patients Who Are Non-Smokers
Source: Nutrients. 2021 Oct 22;13(11):3722. doi: 10.3390/nu13113722 (PMC8619233; doi:10.3390/nu13113722)
Supplement: Supplementary file 1 [file nutrients-13-03722-s001.zip › nutrients-1415851-supplementary.pdf]

Supplemental Table S1. Descriptive statistics and clinical parameters of excluded participants. [1]

	**Included (*n* = 85)**	**Excluded (*n* = 44)**	***P* value**
Age, years	57 ± 11 (34 – 90)	56 ± 14 (23 – 83)	0.57
Sex, n (%)			
Males	42 (49)	17 (39)	0.24
Females	43 (51)	27 (61)	0.24
BMI, n (%)			
Normal, 18.5 – 24.9	18 (21)	16 (36)	0.06
Overweight, 25.0 – 29.9	35 (41)	13 (30)	0.20
Obese, ≥ 30.0	32 (38)	15 (34)	0.69
Smoking status, n (%)			
Never smoked	30 (35)	17 (39)	0.71
Former smoker	33 (39)	12 (27)	0.19
Current smoker	22 (26)	15 (34)	0.33
Baseline clinical measures [2]			
Number of Teeth	25 ± 4 (12 – 32)	25 ± 5 (5 – 32)	0.68
PD, # sites ≥ 4 mm	93 ± 38 (7 – 173)	78 ± 46 (20 – 158)	0.06
BOP, % sites	50 ± 30 (0 – 100)	59 ± 39 (0 – 100)	0.23
Plaque Score, % teeth	72 ± 29 (0 – 100)	72 ± 24 (30 – 100)	0.98
Serum 25-hydroxyvitamin D, nmol/L	61 ± 23 (13 – 129)	56 ± 21 (31 – 132)	0.39

[1] All values are means ± SDs (range) for continuous variables and counts (%) for categorical variables. Non-standard abbreviations: HTN, hypertension; CAD, coronary artery disease; BOP, bleeding on probing; PD, probing depth.
[2] Does not include individuals excluded due to not having ≥ 30% sites with PD ≥ 4 mm.
